# The Effect of a Needs-Related Caries Preventive Program in Children and Young Adults – Results after 20 Years

**DOI:** 10.1186/1472-6831-6-S1-S7

**Published:** 2006-06-15

**Authors:** P Axelsson

**Affiliations:** 1 Department of Preventive Dentistry, Public Dental Health Service, Karlstad, Sweden

## Abstract

The risk for caries development in children varies significantly for different age groups, individuals, teeth, and surfaces. Thus from a cost-effectiveness point of view, caries preventive measures must be integrated and based on predicted risk from age group down to individual tooth surfaces. Based on this philosophy and experiences from continuously ongoing research on evaluating and reevaluating separate and integrated caries preventive measures, as well as methods for prediction of caries risk, a needs-related caries preventive program was introduced for all 0–19-year-olds in the county of Värmland, Sweden, in 1979. The goals for the subjects following the program from birth to the age of 19 years were:

1. To have no approximal restorations.

2. To have no occlusal amalgam restorations.

3. To have no approximal loss of periodontal attachment.

4. To motivate and encourage individuals to assume responsibility for their own oral health.

The effect of the program is evaluated once every year on almost 100% of all 3–19-year-olds in a computer-aided epidemiologic program from 1979. Most of the individualized preventive program was carried out by dental hygienists or prophy dental assistants at clinics in the elementary schools. During the 20-year period the percentage of caries-free 3-year-olds increased from 51% to 97%. In 1999 as many as 86% of the 12-year-olds were caries free. Caries incidence was reduced more than 90% in all age groups. More than 90% did not develop any new caries lesions in 1999. As a consequence, caries prevalence was dramatically reduced. In 12- and 19-year-olds, the mean number of Decayed and Filled Surfaces (DFS) per individual was reduced from 6 to 0.3 and from 23 to 2 respectively. In 19-year-olds the mean number of approximal DFS was <1, and only 0.5 had to be filled. The mean number of occlusal DFS was <1. Since 1995 we have not been allowed to use amalgam in 1–19-year-olds in Sweden. As an effect of our high quality plaque program, approximal attachment loss was prevented, and by efficient education in self-care based on self-diagnosis, needs-related self-care habits were established. Thus it can be concluded that nearly 100% of our goals had been achieved.

## Introduction

According to the World Health Organization's first global caries databank for 12-year-olds, caries prevalence in children from Sweden and some other industrialized countries was among the highest in the world in 1969 [1]. However, during the following decades, caries prevalence in 12-year-olds has been reduced significantly in some industrialized countries – particularly in Scandinavia. The county of Värmland represented the highest caries prevalence in Sweden 30 years ago.

Under the national dental insurance scheme, needs-related dental care, including preventive dentistry, is provided for children up to 20 years of age free of charge, about 95% by the Public Dental Health Service. Sweden's adult population is subsidized by the national dental insurance scheme, yet 60% are treated by private dentists. Sweden has no artificially fluoridated drinking water. Less than 5% of the Swedish population has access to naturally fluoridated drinking water containing >0.7 mg F per litre. In the county of Värmland, less than 2% of the population uses such drinking water.

In 1975, prophy-dental clinics were gradually introduced in elementary schools in the county of Värmland, enabling preventive dentistry assistants or dental hygienists to practice individualized and needs-related preventive dentistry. It is the only province in Sweden with such preventive dentistry clinics in the schools.

In the late 1970s, school-based fluoride mouthrinse programs once every 1 or 2 weeks were still recommended by the Swedish Board of Health and Welfare. However, in populations with high standards of oral hygiene, regular use of fluoride toothpaste, and low caries incidence, the supplementary cariostatic effect of such school programs irrespective of the individual need is questionable. In such populations, individual risk prediction and needs-related combinations of preventive measures are necessary. To ensure a high sensitivity of risk prediction, several etiologic and modifying risk factors have to be combined.

The risk for caries development varies significantly for different populations, age groups, individuals, teeth, and surfaces. Therefore caries-preventive measures must be integrated and based on predicted risk from age groups down to the individual tooth surfaces.

### Goals

Based on this philosophy and on experiences from continuously ongoing research evaluating and re-evaluating separate and integrated caries-preventive measures, as well as methods for prediction of caries risk, a needs-related caries-preventive program for 0- to 19-year-olds was introduced in the county of Värmland in 1979 [2-4]. The goals for the subjects following the program from birth to the age of 19 years were:

1. To have no approximal restorations.

2. To have no occlusal amalgam restorations.

3. To have no approximal loss of periodontal attachment.

4. To motivate and encourage individuals to assume responsibility for their own oral health.

It was hoped that these goals would be attained for 19-year-old participants by 1999. The effect of the program is evaluated once every year on almost 100% of all 3–19-year-olds in a computer-aided epidemiologic program from 1979 [5,6].

## Methods

### Risk age groups

Recent studies have shown that carious lesions appear more frequently at specific ages. This applies particularly to children but can also apply to adults. In children, the key-risk periods for initiation of caries seem to be during eruption of the permanent molars and the period during which the enamel is undergoing secondary maturation.

### Key-risk age group 1: ages 1 to 2 years

Studies by Köhler *et al*. [7,8] showed that mothers with high salivary *mutans streptococci *(MS) levels frequently transmit MS to their babies as soon as the first primary teeth erupt, leading to greater development of caries. Other studies have shown that 1-year-old babies with plaque and gingivitis develop several dental carious lesions during the following years, while babies with clean teeth and healthy gingivae, maintained by regular daily cleaning by their parents, remain caries free [9]. It was also shown that the practice of giving infants sugar-containing drinks in nursing bottles at night increased the development of caries [10].

In another investigation, Grindefjord *et al*. [11] studied the relative risk (odds ratio) that 1-year-old infants would develop caries by the age of 3.5 years. Those with poor oral hygiene, bad dietary habits, salivary MS, little or no exposure to fluoride, and parents with a low educational level or an immigrant background were at 32 times greater risk than were children without the corresponding etiologic and external risk factors. The importance of establishing good habits as early as possible, and of postponing or preventing bad habits, should not be underestimated. In addition, the enamel of erupting and newly erupted primary and permanent teeth is at its most caries-susceptible stage until completion of secondary maturation [12]. In 1- to 3-year-old infants, the specific immune system, particularly immunoglobulins in saliva, is immature. Poor oral hygiene will therefore favor the establishment of cariogenic microflora such as MS.

On average, the permanent teeth in particular erupt 6 to 12 months earlier in girls than they do in boys [13]. On this basis, the first-priority risk age groups are expectant mothers and 1- to 2-year-olds, starting with girls. To prevent postnatal transmission of cariogenic bacteria and poor dietary habits from mother to child, expectant mothers who are at risk should be offered a special preventive program comprising intensified plaque control (mechanical and chemical) and reduction of sugar intake, to reduce the number of cariogenic microflora.

### Key-risk age group 2: Ages 5 to 7 years (eruption of first molars)

Plaque reaccumulation is heavy on the occlusal surfaces of erupting maxillary and mandibular molars, particularly in the distal and central fossae and related fissures. This is in sharp contrast to the fully erupted molars, which are subjected to normal chewing friction [14]. Abrasion from normal mastication significantly limits plaque formation, and this explains why almost all occlusal caries in molars begins in the distal and central fossae during the extremely long eruption period of 14 to 18 months. In contrast, fissure caries is very rare in premolars, which have a brief eruption period of only 1 to 2 months. In addition, the enamel of erupting and newly erupted teeth is considerably more susceptible to caries until secondary maturation is complete, more than 2 years after eruption [12]. Therefore, the caries-reducing effect of fluoride is about 50% greater in erupting and newly erupted teeth than it is in teeth that have undergone secondary maturation.

The next high-risk age is, therefore, from 5 to 7 years, during eruption of the first molars (the key-risk teeth), starting with girls. Intensified mechanical plaque control twice a day with fluoride toothpaste should be performed by the children's parents, particularly on the erupting first molars. Home care should be supplemented at needs-related intervals by professional mechanical tooth cleaning (PMTC) and fluoride varnish. In the most caries-susceptible children, resin-modified glass ionomer cement should be used in the fissures, as a slow-release fluoride agent.

### Key-risk age group 3: Ages 11 to 14 years (eruption of second molars)

Normally, the second molars start to erupt at the age of 11 to 11.5 years in girls and at around the age of 12 years in boys. The total eruption time is 16 to 18 months. During this period, the approximal surfaces of the newly erupted posterior teeth are undergoing secondary maturation of the enamel and are also most susceptible to caries. Therefore, 11- to 14-year-olds have not only, by far, the highest number of intact tooth surfaces, but also the greatest number of surfaces at risk. Integrated plaque-control measures and use of fluoride agents should therefore be intensified on the approximal surfaces of all the posterior teeth and the occlusal surfaces of the second molars, starting with 11- to 11.5-year-old girls, to protect intact tooth surfaces and to remineralize non-cavitated carious lesions. If this program is maintained throughout the secondary maturation period, and needs-related self-care habits are established, there is a high probability that the remaining intact tooth surfaces will remain intact for the individual's entire life.

#### Key-risk individuals

In children, caries prevalence and caries incidence related to the age group and the combination of Plaque Formation Rate Index (PFRI) (score 0–5, by Axelsson [15] based on 24-hour de novo plaque reaccumulation, plus salivary mutans streptococci (MS) levels) – will give the highest sensitivity value for prediction of caries risk [15,3]. The percentage of selected key-risk individuals should also be related to age. In other words, the highest percentage of key-risk individuals should be selected from the 11–14-year-olds and the lowest percentage in 3–4, 8–10, and 15–19-year-olds.

An analysis of caries prevalence (mean DFS in >650 14-year-old children) related to different PFRI scores indicated a threshold for caries risk between PFRI scores 2 and 3. This was subsequently confirmed in the longitudinal part of the study, over 5 years [15]. For MS this critical level was between 0 and 100, 000 CFU/ml saliva. In the longitudinal part of the study, MS-positive individuals with PFRI scores 3–5 developed 5 times more approximal dentin carious lesions per individual per 5 years than MS-negative individuals.

Based on the experiences from these studies, the following guideline for selection of no caries risk (C0), low caries risk (C1), caries risk (C2), and high caries risk (C3) by the combination of MS-salivary test and PFRI was designed. A salivary MS test screens out MS-negative subjects (about 20%) as not being at risk. Of the remaining 80% or so (SM-positive subjects), those with a PFRI ≥ score 3 are selected as risk patients (approximately 25%). From these subjects, an extremely high-risk group may be further selected: those with a PFRI score 4 or 5 and MS >1 million CFU/ml saliva (around 5%).

More detailed criteria for selection of C0, C1, C2, and C3 individuals in different age groups based on caries prevalence, caries incidence, etiological factors, external and internal modifying factors, and preventive factors are presented by Axelsson [3].

#### Key-risk surfaces

Depending on the age and caries prevalence of the population, there may be pronounced variations in the pattern of both lost teeth and decayed or filled surfaces. The molars are clearly the key-risk teeth. In a toothbrushing population, the key-risk surfaces are the fissures of the molars and the approximal surfaces, from the mesial aspect of the second molars to the distal aspect of the first premolars. Integration of mechanical plaque control by self-care and the use of fluoride toothpaste, supplemented at needs-related intervals by PMTC, fluoride varnish, and chlorhexidine varnish should therefore target these key-risk teeth and surfaces.

The risk for development of approximal carious lesions seems to be correlated to the buccal-lingual width of the tooth crown – particularly in toothbrushing populations. That is because of the limited accessibility of the toothbrush on the wide approximal surfaces of the molars and premolars, which are blocked by buccal and lingual papillae.

#### Preventive programs

##### 0–2-year-olds

Dental hygienists or preventive dentistry assistants provide antenatal counseling on an individual and group basis. To prevent postnatal transmission of cariogenic microbes and poor dietary habits from mother to child, selected at-risk individuals are offered a special preventive program at public dental health centers. At the child welfare centers, dental hygienists or preventive dentistry assistants counsel parents on good oral hygiene and dietary habits for their children, as well as the importance of early introduction of the use of fluoride toothpaste. A pea-sized amount of fluoride toothpaste is recommended on the toothbrush in order to reduce side effects of swallowed toothpaste. However, no systemic fluorides (tablets etc.) were used. The overall goals in these age groups are to establish good habits as early as possible and to postpone bad habits as long as possible.

##### 3–5-year-olds

In kindergarten, preventive dentistry assistants or dental hygienists carry out a preventive program, with the teachers' assistance. The preventive program includes supervised toothbrushing with a fluoride toothpaste, and games based on oral health education. Special efforts are focused on education of the parents to be responsible for the daily cleaning of their children's teeth. The selected at-risk individuals (about 10%) receive PMTC and fluoride-varnish treatment 2–4 times per year.

##### 5.5–7.5-year-olds

In this age group the caries preventive methods are focused on maintaining the fissures of the first permanent molars caries free until the teeth are fully erupted and exposed to normal functional chewing wear. Thereafter the risk for development of occlusal caries should be over. Therefore the parents are educated to observe when the first molars start eruption and how to intensify cleaning the fissures twice a day with a special toothbrushing technique and fluoride toothpaste. As soon as the mesial surfaces of the first molars are in contact with the distal surfaces of the second primary molars, the parents are responsible for daily cleaning of these surfaces with fluoride dental tape in a special holder. Based on the predicted risk, the daily toothbrushing performed by the parents is supplemented with needs-related intervals of PMTC, use of fluoride and chlorhexidine varnishes and fissure sealants with light-cured resin-modified glass ionomers as a slow-release fluoride agent during the eruption of the molars.

##### 8–11.5-year-olds

In this relatively low-risk age group, the children are educated by dental assistants or dental hygienists in the preventive clinics of the elementary schools and at the public dental health clinics on how to gradually take over the responsibility from their parents for the daily cleaning of their own teeth. About 10% are selected at-risk individuals who may receive supplemented needs-related professional caries preventive treatment.

##### 12–14-year-olds

This age group is the key-risk age group and should receive the most generous and intensive caries preventive program from a cost/effectiveness point of view. They have the highest number of still caries-free but highly caries-susceptible permanent tooth surfaces until the second molars are fully erupted and the so-called secondary maturation of the enamel is achieved.

Dental hygienists or preventive dentistry assistants give lessons on preventive dentistry, as well as self-care education, in the elementary schools. They cooperate with schoolteachers, school nurses, dietary consultants, psychologists, school physicians, and dentists as a health team in order to optimize oral health as well as general health from a "holistic" point of view. For example, our dental hygienists and prophy dental assistants have been pioneers in successfully preventing the debut of smoking in children from the age of 12 years.

Based on the experiences from a Brazilian study [16], needs-related oral hygiene habits were established particularly from the age of 12 years. Starting with girls from the age of 11.5 years, specialized education was focused on the cleaning of the fissures of erupting second molars. In particular, 12–13-year-olds were given careful education in applying fluoride dentifrice on dental tape to clean the approximal surfaces of the molars and premolars prior to brushing. The PFRI score indicated how frequently the teeth should be cleaned and which teeth should be cleaned first with special attention to surfaces with heaviest plaque re-accumulation. For individuals with PFRI score 4–5 and high caries risk, it was recommended that they clean their teeth just before each meal and take a fluoride chewing gum straight after the meal.

Based on the predicted caries risk, supplementary professional caries preventive measures such as PMTC, topical application of fluoride varnish, chlorhexidine varnish, and fissure sealants with resin-modified glass ionomers (as a slow-release fluoride agent in erupting second molars) were performed by dental hygienists or prophy dental/assistants with needs-related intervals at the preventive clinic in the schools or at the public dental health clinics.

##### 15–19-year-olds

In this age group, needs-related self-care habits should already have been established and the so-called "secondary maturation" achieved. Thus these ages are regarded as a low-risk group. However, special attention must be focused on erupting third molars in similarity with the problems related to erupting first and second molars. In addition it must be observed that an "unhealthy lifestyle," such as bad dietary habits, etc., may occur in some individuals who leave home early to study in cities away from their homes.

### Caries diagnosis and evaluation

Caries diagnosis was carried out by calibrated dentists in the public dental health service according to the Swedish Board of Health and Welfare's recommendations for collecting epidemiological data. Buccal and lingual surfaces showing caries lesions with cavitaton by probing were regarded as DS (Decayed Surfaces). Caries lesions on the approximal surfaces of the molars and premolars and the occlusal surfaces of the molars were diagnosed by bitewing radiographs, as well as by probing. All lesions in dentin diagnosed on radiographs were regarded as DS even without clinically diagnosed cavitations. For comparison, the caries diagnosis criteria by WHO for national epidemiological surveys are based only on clinically visible cavities and by probing. All our filled surfaces are regarded as FS. Missing surfaces are not included. That is because no teeth are missing because of caries in the permanent dentition of our 6–19-year-olds. (Missing teeth have been extracted for orthodontic reasons only.)

Since 1979, the effects of our needs-related preventive programs have been evaluated annually in almost 100% of 3–19-year-olds at surface-, teeth-, individual-, clinic- and county levels in a computerized system.

## Results

### Caries prevalence in primary teeth

As an effect of the preventive program we gradually introduced at the health centers for expectant mothers and the child welfare centers in the 1970s, the percentage of caries-free 3-year-olds increased from 35% in 1973 to 97% in 1993 and is still maintained at this suboptimal level. In 1999 the mean values of deft (decayed, extracted or filled teeth) in 3-, 4- and 5-year-olds were only 0.07, 0.3, and 0.4, respectively. The percentages of caries-free 3-, 4-, 5-, and 6-year-olds were 97, 90, 83 and 76% respectively.

### Caries prevalence in permanent teeth

**Figure 1:**
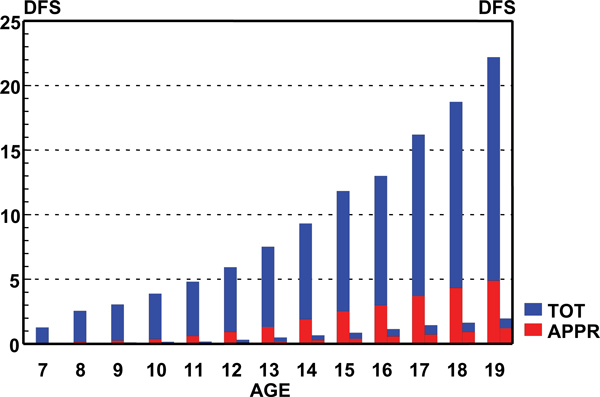
Caries prevalence 1979 and 1999. County of Värmland.

**Figure 2:**
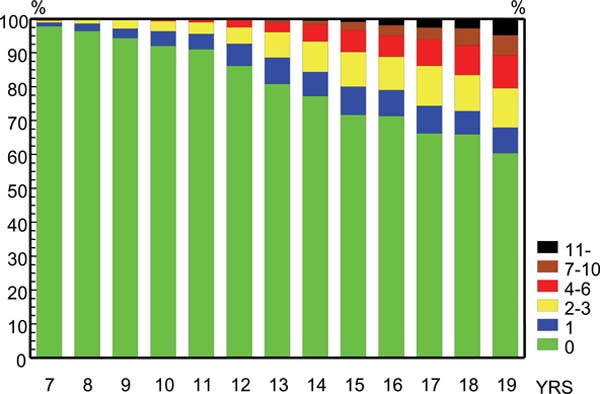
Caries prevalence, County of Värmland 1999. DFS frequency distribution.

Fig. 1 presents caries prevalence in the county of Värmland expressed as mean DFS, all surfaces and approximal surfaces per individual in 1979 and 1999, in all age groups from 7–19-year-olds. The average caries reduction ranged from 85–95%. The mean total number of DFS per individual aged 12, 16, and 19 years respectively, declined from 6, 12, and 24.3 DFS in 1979 to 0.3, 1.15 and 2.1 DFS in 1999. In the same age groups, DFS on the approximal surfaces representing 20–25% of the total in 1979, declined from 1.1, 3.0 and 5.0 in 1979 to 0.1, 0.6 and 1.1 DFS in 1999. The frequency distribution of DFS in the different age groups in 1999 showed that the percentages of caries-free individuals from age 7 to 19 years were 98%, 96%, 94%, 92%, 91%, 86%, 81%, 77%, 72%, 71%, 66%, 64% and 60%, respectively (Fig 2).

### Caries incidence in permanent teeth

**Figure 3:**
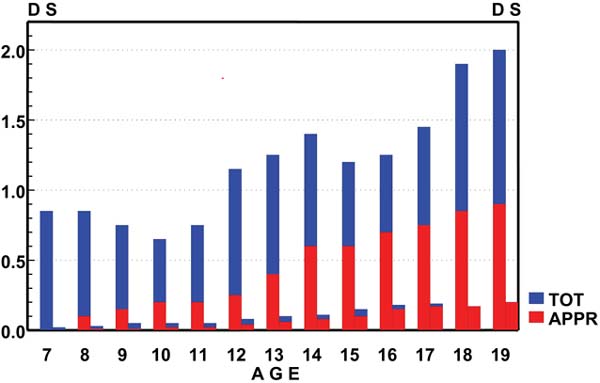
Caries incidence 1979 and 1999. County of Värmland

**Figure 4:**
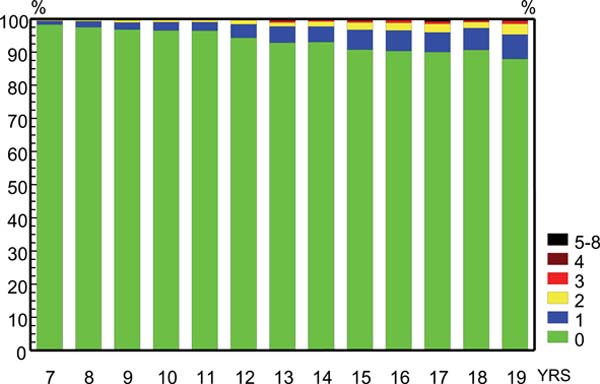
Caries incidence in Värmland 1999. DS-TOT frequency distribution

Compared to 1979, the mean number of new DSs per individual per year was also reduced between 85% and 95% in 1999, including all surfaces, as well as the approximal surfaces, in the different age groups. In 7-, 12-, and 19-year-olds, the caries incidence for all surfaces dropped from 0.85, 1.15, and 2.0 new DSs per individual in 1979 to 0.02, 0.06, and 0.2 in 1999 (Fig 3). In the same groups, caries incidence for the approximal surfaces declined from 0, 0.2, and 0.9 new DSs per individual in 1979 to 0, 0.03 and 0.2 in 1999. The frequency distribution of new DSs showed that 88–98% of the children in the different age groups did not develop any new DSs in 1999 (Fig 4). Among 7-year-old children, 98% did not develop any new DSs. 1% developed one new DS, and 0.4% developed 2 new DSs. The corresponding figures for 12-year-olds were 94%, 4%, and 1% respectively. Among 19-year-old individuals 88% developed no new DSs, 8% had one new DS, 3% had two new DSs, 1% had three or four new DSs, and 0.1% had five or six new DSs.

### Treatment time and cost effectiveness

According to data collected annually from all counties in Sweden by the Swedish Board of Health and Welfare, the mean treatment time by a dentist per child in 1979 was 1.75 hours in the county of Värmland, and the national average was 1.7 hours. However, as an effect of our needs-related preventive program by preventive dental assistants and dental hygienists, the mean treatment time by a dentist was dramatically reduced to only 20 minutes per child per year in 1999, which is the outstanding lowest value among the Swedish counties. Most of the dentist's time was spent on examinations, because the need for restorations was minimized. The total costs per child per year (2001) including the needs-related preventive program by dental hygienists or preventive dental assistants, treatment by dentists, and orthodontic treatment by specialized orthodontists was about 90 euros (US $120) compared to 100 euros (US $135) as the average for all Sweden. Because the average caries prevalence in children and young adults is the lowest in Sweden, it must be concluded that this needs-related caries preventive program is very cost effective. The cost/benefit ratio of such a program is also very high because intact teeth and healthy gingiva are beautiful, functional, appealing, and may positively influence our general health. As a contrast, unreasonable "drilling, filling, killing the pulp, and billing" should have gone out of fashion many decades ago.

#### Nearly 100% of our goals were achieved and we still improve our results

Caries prevalence in 19-year-olds was reduced from more than 24 DFS in 1979 to only 2 DFS in 1999. Out of these, one DFS was occlusal. Since 10 years ago it is not allowable to use amalgam in Sweden on 1–19-year-olds. Therefore very few occlusal amalgam fillings exist because of caries in the 19-year-olds, and in a few years none will exist. On average, there was only one approximal DFS in 1999 and less than 0.5 was filled, as included non-cavitated dentin carious lesions diagnosed on bitewing radiographs should be arrested by "prevention instead of extention" or at least "prevention before extention."

Needs-related self care habits based on self-diagnosis were established as early as possible. In addition, PMTC and chemical plaque control were supplemented in at-risk individuals at needs-related intervals. As a consequence, approximal loss of periodontal attachment was prevented. Thus, it can be concluded that close to 100% of our goals 20 years ago have been achieved. In addition it seems realistic that 20-year-olds, with no lost teeth, only 2 DFS, no loss of periodontal attachment, and well-established excellent self-care habits should maintain at least 25 healthy natural teeth for life. This seems realistic, as we already have proven in our 30-year longitudinal study in adults. In that study, 51–65- and 66–80-year-olds who participated in a needs-related preventive program performed by a dental hygienist from 1972 to 2002 on average lost only 0.4 and 0.7 teeth respectively, did not loose periodontal attachment, and developed only 2 new DSs per subject per 30 years [4].

## Conclusion

As a matter of fact, we are still taking action to reduce caries prevalence. We continue our clinical research on improving and testing new preventive materials and methods; we continue implementing our methods to predict risk for oral diseases; we pursue new knowledge from others; and we provide continued education for our well-trained and highly motivated dental professionals. Because of these actions, it seems reasonable that caries reduction in our children and young adults can continue to be improved at an even lower cost. Finally, the importance of evaluating new methods must be emphasized, as well as re-evaluating established methods by clinical research in our own population before large scale implementation (in contrast to the hazards of direct implementation from animal and in vitro experiments). There are "evergreens," but preventive measures should be tailored to reflect trends in the pattern of dental disease in a population.

## Competing interests

The author(s) declare that they have no competing interests.
